# 832 Continuous Real-Time IAP Monitoring in Severe Burns: A Case Study

**DOI:** 10.1093/jbcr/iraf019.363

**Published:** 2025-04-01

**Authors:** Jana Alley, Patrick Beer

**Affiliations:** Advent Health; Hurley Medical Center

## Abstract

**Introduction:**

Managing severe burns often necessitates aggressive fluid resuscitation, which can lead to complications like intra-abdominal hypertension (IAH) and abdominal compartment syndrome (ACS). Traditional intermittent intra-abdominal pressure (IAP) monitoring may not always capture critical pressure changes, underscoring the need for continuous monitoring in high-risk patients.

**Methods:**

We present the case of a 28-year-old male with 49% total body surface area burns, treated with Parkland formula-guided fluid resuscitation and early surgical debridement. Continuous IAP monitoring was implemented using a specialized urethral catheter with an electronic transducer. IAP levels were tracked in real-time alongside mean arterial and abdominal perfusion pressure.

**Results:**

On the second day post-admission, IAP spiked to 20 mmHg, signaling an increased risk of ACS. Immediate intervention reduced the IAP to under 10 mmHg within 24 hours, effectively preventing ACS without requiring invasive procedures.

**Conclusions:**

This case highlights the potential of continuous IAP monitoring as a valuable tool in severe burn management. Early detection of IAH allowed for timely intervention, reducing the likelihood of ACS and potentially lowering patient morbidity. Continuous monitoring may suggest a new standard of care in high-risk burn patients, although further research with larger cohorts is necessary to validate these findings.

**Applicability of Research to Practice:**

Continuous IAP monitoring shows promise in improving outcomes for high-risk burn patients, offering a non-invasive early detection and intervention method.

**Funding for the Study:**

N/A

